# More of the same? Israel’s expanded carrier screening for cystic fibrosis

**DOI:** 10.1038/s41431-025-01851-8

**Published:** 2025-04-25

**Authors:** Tamar Nov-Klaiman, Ruth Horn, Aviad Raz

**Affiliations:** 1 Institute for Ethics and History of Health in Society (IEHHS), Faculty of Medicine, University of Augsburg, Augsburg, Germany; 2 Department of Sociology & Anthropology, Ben‐Gurion University of the Negev, Beer‐Sheva, Israel

**Keywords:** Ethics, Health policy

In late 2024, Israel’s national genetic carrier screening program was significantly expanded [1]. It now includes 650 pathogenic variants in 290 genes, offered to the entire population and funded by the Ministry of Health. In the case of cystic fibrosis (CF), 70 variants associated with the disease are now being tested for, compared to 18 CF-causing variants before the expansion. This commentary discusses possible ethical and social implications of the expanded screening panel for CF.

CF is an autosomal recessive disorder and known as being the most common life-limiting genetic disease among individuals with European ancestry [2]. The Israeli CF carrier rate is 1:49 nationally, though it gets as high as 1:29 in the Ashkenazi Jewish population [3]. The disease is caused by pathogenic variants in the CF transmembrane conductance regulator (CFTR) gene, in which over 1000 CF-causing variants have been identified (CFTR2 database (https://cftr2.org)).

Two models of screening exist in relation to CF: newborn screening (NBS) and population genetic carrier screening (PGCS). The majority of countries with high CF prevalence—including Australia, North America, and many European countries—have established NBS programs [2], with roughly 10 million newborn babies screened annually for CF worldwide [4]. Less commonly used is the model of a national population-wide carrier screening, of which Israel is a world leader. Several other countries, such as the US, offer CF carrier screening and recommend it to the general population [4].

Since 2008, CF is included in the Israeli national genetic carrier screening program offered to the whole population and funded by the Ministry of Health. Of note, Israel has a NBS program for the early detection of 13 endocrinological, metabolic and immunological conditions, e.g., phenylketonuria, congenital hypothyroidism and severe combined immunodeficiency. The program is offered to the entire population free of charge and is performed within 36–72 h after birth [5, 6]. However, despite former attempts by CF physicians and lobbying by the Israeli CF Foundation, NBS for CF is not available in Israel. The argument put forward for this decision is the performance of the PGCS program resulting in relatively small numbers of babies born with the condition [6, 7]. Thus, Israel is a unique case of a country that has markedly been prioritizing PGCS over NBS for CF. In contrast, most other countries chose not to screen carriers, initially due to the Wilson and Jungner Criteria [8]—to screen only if you can treat—since in the case of PGCS, the aim is not treatment in the traditional sense, but rather an intervention that involves reproductive autonomy [9]. While the treatment principle remained largely intact in the context of NBS for a long time, it was expanded in other contexts to enable screening that could provide other forms of public benefit, such as to inform reproductive decisions and provide knowledge of the incidence of a condition. Indeed, scholars claim that the Wilson and Jungner principles are not compatible with carrier screening, where other criteria are relevant in policymaking and implementation [9]. Moreover, while carrier screening programs may not necessarily facilitate treatment in the conventional way, for some conditions such as CF—for which treatment is available—PGCS may facilitate early treatment in the future offspring of identified carrier couples.

Carrier screening in Israel is performed before or during pregnancy in a sequential manner, with the woman most often being tested first. If she is found to be a carrier, the partner is then tested. If both partners are detected as carriers, they receive genetic counseling and may choose between several reproductive options free of charge. These include prenatal testing (chorionic villus sampling or amniocentesis) or preconception genetic testing (PGT) following in vitro fertilization (IVF) [6]. While accurate data regarding the uptake of PGCS is lacking, it is estimated that a very high proportion of families in the general population perform PGCS [3].

During the years 2008–2018, the Israeli policy allegedly resulted in a dramatic decrease in the birth rate of babies with CF (to around 1 in 200,000 in 2018); a shift toward milder phenotypes; and marked increase in PGT uptake by carrier couples alongside constant CF pregnancy termination rates [3].

In the literature and international debates, selective reproduction is often contested on ethical, religious and social grounds such as the moral status of a fetus or the risk of discrimination against people with a particular condition that is screened for. However, the empirical evidence of discrimination as a consequence of selective reproductive decisions is weak, and the superiority of the moral status of the fetus over reproductive autonomy is debatable [10, 11].

Indeed, global accounts of the aim of prenatal and carrier screening programs by official bodies reflect a rhetorical shift that has occurred over the years—from a ‘prevention paradigm’, where prevention refers to the avoidance of a disease and the reduction of its prevalence through selective reproduction, to an ‘autonomy paradigm’, where informed reproductive decision-making is the stated goal [12].

While the Israeli policy broadens women’s reproductive leeway via a wide array of preconceptional and prenatal tools, the decision to embrace exclusively carrier screening while forgoing NBS for CF has also negative medical, ethical and social implications. The current policy harms babies affected by CF who were missed by the carrier screening program—either because of their parents not making use of it or because the variants involved are rarer and therefore not included in the panel used. Of note, the utilization of PGCS is considerably lower in the Arab and Jewish orthodox populations [3]. In the absence of NBS, the diagnosis and following treatment are delayed, resulting in worse outcomes for the patients [3].

Dotan et al. [3] claimed in light of the outcomes of the former 18-variant panel, that such a low incidence of CF births “raises the question of whether CF NBS is still appropriate” [3]. This view could be accentuated by the recent expansion of the testing panel, which would arguably further reduce the incentive to implement NBS and make it harder to justify, since fewer babies with CF will be born. However, with a lower disease prevalence and with physicians increasingly relying on PGCS for effective detection in the population, there is higher risk of CF being overlooked when clinicians are presented with a sick infant, resulting in delayed diagnosis and treatment and worse outcomes. This is more likely to affect communities in which PGCS uptake is lower.

From the perspective of the patients, the carrier screening expansion could be justified on the premise of giving couples more actionable information that could be used according to their values and preferences, thereby supporting their reproductive autonomy. It allows those who so wish to prevent the birth of a child with CF and the associated difficulties that may arise for the future child and family members.

From the perspective of the medical establishment, a utilitarian rationale—i.e., cost-benefit calculations—could justify the move. This rationale is arguably more prominent in recent years, in the presence of the new and expensive treatments (CFTR modulators) that—depending on the patients’ genotype—have the potential to dramatically improve their quality of life and extend life expectancy [4]. Since these drugs would need to be administered throughout patients’ lives, their costs are considerable [4]. These drugs are publicly funded in Israel for patients with a genotype proven to respond to the treatment and involve substantial costs for the healthcare system.

The new treatments, therefore, arguably motivate opposite policies. On the one hand, they offer (some) patients a significantly improved prognosis, thereby potentially shifting the balance towards care and increased quality of life, rather than prevention. These therapies are becoming available at earlier ages, thereby underscoring the importance of NBS as facilitating early diagnosis and treatment. However, they present significant additional costs for the healthcare system and might thereby incentivize a policy which enables prevention of the disease (through carrier screening and selective reproduction) rather than care. As such, expensive treatments may be used as a reason both in favor of and against carrier screening and—by implication—NBS.

Israel’s PGCS program—especially in its new form—is unique, both in terms of the scope of the diseases for which it tests and in terms of state funding for the screening itself and for subsequent services (prenatal testing/IVF and PGT). Israel has been long known for its unique stance on genetics services, attributed to a variety of cultural, political, and professional grounds [13]. Genetic testing and selective reproduction receive wide support from the public, including among patients, families, and disability rights activists [14, 15]. Moreover, Israeli physicians conceptualize genetic screening as a form of preventive medicine, and the aim of prevention of severe genetic conditions is rooted in the medical community handling pregnancy [6]. Raz (2018) argued that in its genetic policies, “Israel is both reckless and pioneering—depending on one’s perspective” [13]. The early adoption of such technologies in Israel could lead the way for other countries. Indeed, a recent pilot—the Mackenzie’s Mission project, funded by the Australian government—experimented with carrier screening of more than 9000 reproductive couples, encompassing over 1280 disease-associated genes, to inform a potential government-funded carrier screening program [16]. Of note, publicly funded NBS and carrier screening for CF are already implemented in Australia.

While a routinized offer of PGCS is aimed at promoting reproductive autonomy and equitable access, it is also claimed to have possible negative implications, e.g., societal pressure to partake in screening, stigmatization of people or communities who decline it (especially when it leads to affected children), and discrimination of people living with the conditions screened [11]. However, an extensive review study on the societal implications of expanded universal carrier screening found no empirical evidence of such stigmatization and discrimination [11].

Finally, it is important to recognize that even a well established PGCS program does not guarantee detection of all affected pregnancies [17]. As long as there are pathogenic variants that are not included in the testing panel, and given that PGCS uptake is not 100%, NBS and PGCS have a complementary nature. Together they facilitate both reproductive autonomy and a better outcome for those born with the condition [17].
